# Glass Ionomer Cement as a preventative fissure sealant for first permanent molars in high caries risk patients waiting general anaesthetic—a case series

**DOI:** 10.1038/s41405-022-00119-3

**Published:** 2022-09-03

**Authors:** Toby Andrew Mummery, Riddhi Popat

**Affiliations:** Harrogate and District NHS Foundation Trust Community Dental Service, North Yorkshire, UK

**Keywords:** Fissure seal, Dental treatment planning, Paediatric dentistry, Glass-ionomer cement

## Abstract

**Objective/aim:**

The aim of this case series was to assess the ongoing suitability of Glass Ionomer Cement Fissure Sealants for use in paediatric patients. These had been used through the COVID pandemic due to their status as a non-aerosol-generating procedure.

**Materials and methods:**

A retrospective clinical review was undertaken to identify cases where GIC Fissure Sealants were used in paediatric patients awaiting exodontia general anaesthetic within Harrogate and District NHS Foundation Trust Community Dental Service. Identified cases were then collated to form this case series. These were reviewed regarding the number of GIC fissure sealants placed, and retention at the GA appointment and any subsequent follow-up appointments.

**Results:**

The results showed favourable results of GIC fissure sealants, with an average retention of 77% – in line with the wider literature results for GIC fissure sealants.

**Discussion:**

The results showed a favourable retention rate, particularly given patient challenges leading to them requiring exodontia GA. Review of alternative fissure sealant materials may be beneficial on a local level to compare results with the available literature and confirm ongoing suitability.

**Conclusions:**

The results appear to support the ongoing use of GIC-FS where close patient monitoring is available.

## Introduction

### Background

Dental caries is a highly prevalent non-communicable disease with a multifactorial aetiology [1]. Research shows that it can be prevented and even reversed in it’s early stages [2]. Fissure sealants provide a physical barrier and block nutrition to prevent biofilm growth. Resin based fissure sealants (RB-FSs) have an excellent and long-standing evidence for providing long term protection for fissures, particularly first permanent molars [3]. Current guidelines recommend the use of RB-FSs for moderate to high risk patients [4].

RB-FSs require acid etch prior to placement [5]. As such, removal of the etch is a necessary clinical step in their placement. The evidence base shows that full clearance of the etch requires extended use of the 3in1 [6] with this being considered by the Chief Dental Officer and Public Health England to produce an aerosol [7]. As such, under guidance regarding cross infection control during the SARS-COV-2 pandemic this required fallow time and had a significant impact on overall service provision.

Glass Ionomer Cement (GIC) has been identified as an alternative for RB-FSs in situations where moisture control is poor [8]. This is particularly true for partially erupted 6s in high caries risk children [7] and has the advantage of some limited fluoride release [5]. The main limitations of GIC fissure sealants (GIC-FSs) include reduced retention compared to resin-based sealants [5].

Harrogate and District NHS Foundation Trust Community Dental Service (HDFT-CDS) provides Community Dental Care to patients throughout the North Yorkshire area.

### Rationale

Due to the limitations of AGPs during COVID-19 restrictions, and the accessibility of GIC as an alternative FS material, an increase in the use of GIC was anecdotally observed within HDFT-CDS over the pandemic.

A service evaluation was developed to assess the efficacy (as measured by retention) of GIC-FSs, with the view to informing ongoing practice.

Clinical retention has long been used as a measure to assess the effectiveness of fissure sealants. A service evaluation was developed to assess the efficacy (as measured by retention) of GIC-FSs, with the view to informing ongoing practice. HDFT-CDS sees patients that have complex and medical social needs, and such, GIC-FSs would be valuable material options should they prove to be comparable to RB-FSs.

### Aims and objectives

The aim of the service evaluation was to determine the retention of GIC-FSs placed in children undergoing exodontia general anaesthetics (X-GA) in HDFT-CDS.

Objectives included:

1. to review X-GA sessions to identify patients in which GIC-FS had been placed,
2. to identify the time elapsed between placement and review (GA or clinic review),
3. to identify the number of GIC-FS placed, and their retention at GA or clinic review.

## Materials and methods

A retrospective service evaluation was developed to assess the retention of GIC-FSs being placed prior to X-GA.

Inclusion criteria were: seen on Exo-GA list, 6s erupted, GIC-FS charted as placed prior to GA. These were chosen as children undergoing GA for dental extractions would almost universally fall into an increased risk category, making them suitable candidates for FSs [4]. The presence of 6s and the explicit use of GIC as the material mapped to the stated aims and objectives. Exclusion criteria were: existing GICs prior to assessment in HDFT-CDS. These were chosen to allow direct comparison of retention rates from the point of placement.

Additional demographic information (e.g. ethnicity, IMD of postcode, etc) were not considered to add to the results and hence were not collected.

A notes review was undertaken with a view to establishing the total possible sample. This spanned Sept-2020 to Jan-2022 and was reflective of the period during which service provision was affected by limitations from COVID. A total of 20 cases meeting the inclusion criteria were identified. A sample size calculation (sample size = ((*z*^2xp(1−*p*))/e^2)/(1 + ((z^2xp(1−*p*)/e^2 N))) where *N* = population size, *e* = margin of error, *z* = z-score) was undertaken which returned a sample size of 20 (i.e.100%).

Information collected included: age (whole years), number of GIC-FSs placed, time between placement and GA (months), number of GIC-FSs lost by GA, time between GA and review (if appropriate), number of GIC-FSs lost by review (if appropriate).

Simple statistical analysis was run including mean, median, mode, range used to interpret the results.

### Ethics

This service evaluation reviews treatment outcomes of a well-established treatment modality within the local setting. As such, it was felt it was not testing a hypothesis or adding significantly to the existing evidence base. This was confirmed utilising the Health Research Authority tool for assessing the need for Research Ethics Committee review [9].

Given patient identifiable information was not collected or presented, it was also confirmed that explicit individual patient consent for access to their records, collection, or interpretation of results was not required.

Local measures to ensure ethical practice included:

1. Anonymisation of patient information. This included the recording of age in whole years, and not recording the specific date of a patient’s general anaesthetic alongside any clinical information.
2. Data security. This included the recording of all information on trust systems and the limited dispersal of fully anonymised/averaged data beyond this.

## Results

The review period spanned from Sept-2020 to Jan-2022.

A total of 20 patients were included. Collated results can be seen in Table 1, representing a total of 61 GIC-FS. Between placement and GA (average of 5.35 months), a retention of 79% was observed (13 failures in ten patients). Three patients were reviewed by HDFT-CDS, with the remaining 17 discharged to GDP. For the three patients followed up a total of eight GIC-FS were reviewed. Between GA and clinical review (average 6.67 months), a retention rate of 92% was seen (one failure in one patient).

**Table 1:** Average retention of GIC-FSs at follow-up (FU).

	Age of patient (Yrs)	Number GIC FS placed	Time between placement and GA (months)	Retention	Time between GA and FU (months)	Retention	Total Time between placement and FU (months)	Total Retention
Mean	7.20	3.05	5.35	0.79	6.67	0.92	6.35	0.77
Median	7.00	4.00	6.00	0.88	6.00	1.00	6.50	0.75
Mode	7.00	4.00	3.00	1.00	6.00	1.00	6.00	1.00
Range	6–9	1–4	1–11	0–1	6–8	0.75–1	1–11	0–1

## Discussion

The retention of pit and fissure sealants has long been used as a measurement of their caries preventative rates, and therefore success. Almost double the studies assessing the success of fissure sealants report retention as an outcome compared to those that report dental caries [10]. It is believed that full retention of the sealant material provides an effective barrier and seals cariogenic microorganisms from the oral environment, thus preventing the development of dental caries [10]. A Cochrane review concluded that there is insufficient evidence to judge the relative effectiveness of GIC-FS and RB-FSs [3].

There was no existing data regarding number or retention of RB-FSs within HDFT-CDS so comparisons were drawn to existing literature and evidence base [5, 11]. Although moisture control for GIC-FS isn’t as imperative as it is for RB-FS [12], the hydrophilic GIC still requires some element of isolation. Prior to their second stage of setting, GICs are weak and soluble, and therefore require protection from excessive moisture contamination [12]. Ideal conditions may be difficult to replicate with some of the patients seen by HDFT-CDS. Furthermore, the low wear strength of GIC affects their ability to withstand occlusal forces [12]. The published retention for RB-FS at 6 months is around 91% and the published retention for GIC-FS at 6 months is around 78% [11]. Given the remit of HDFT-CDS (i.e. patients unable to be treated in general practice due to behavioural or medical barriers to care) it is feasible that it is more difficult to obtain ideal circumstances for FSs, and hence a lower retention rate may be expected [13].

The retention of the GIC-FS placed within HDFT-CDS was comparable to the rates published in literature at 6 months’ follow up. Most of the failures occurred within the first 6 months of placement. Given the significant limitations to the provision of care during the pandemic, this arguably supports the use of GIC-FSs and an interim preventative strategy.

GIC-FS are clinically faster to place than RB-FS. The average RB-FS takes 10.56 min to be applied to all first permanent molars on a paediatric patient [14]. As GIC-FS has less stages, and less equipment, they require less chair time. As of March 2022, discounting operator costs and chair costs (which were significantly higher during the pandemic due to increased cleaning requirements and reduced clinical availability), it costs around £4.70 to place GIC-FS on four permanent molars, for a single patient, and around £6.65 to place RB-FS [15]. Other studies have also concluded that GIC-FS are a cheaper alternative in dental outreach settings [16]. The efficiency of the GIC-FSs is very useful for some of the patients being seen by HDFT-CDS. However, studies show that GIC-FSs require close clinical review, to ensure that there is no development of disease, especially if biofilm control is less than ideal [17]. We would expect to see the high-risk patients that fissure sealants are applied to every 3 months, as per NICE guidelines [18, 19].

As high-risk patients, it is expected that many of the patients included in this case series would have topical fluoride applied, either by their general dental practitioner or by a clinician in HDFT-CDS. Whilst there is low certainty evidence that placing RB-FS as well as applying fluoride varnish alone reduces caries by 77% [20], there is little research regarding the efficacy of GIC-FS and fluoride varnish. This is beyond the remit of this case series.

### Strengths

The findings of this informal case series are supported by the existing evidence regarding retention rates. Given the remit of HDFT-CDS it is arguably more relevant to this patient base (where aspects such as patient management and moisture control are more challenging) than a general practice environment where RB-FSs may be more achievable. It further supports the existing, higher-quality publications that GIC-FS are an acceptable and appropriate alternative where RB-FSs is not achievable.

Fissure sealants have also been used as a treatment option for the management of initial occlusal caries into dentine. Sealing in caries slows or arrests the progression of the lesion developing and reduce the risk of a permanent molar or premolar requiring a restoration. Guidelines recommend the use of RB-FS but acknowledge the use of GIC-FS as a temporary sealant or restoration if there is insufficient cooperation [21]. Again, this is something that would be beneficial for the cohort of patients seen within HDFT-CDS.

### Limitations

The lack of local data regarding the retention of RB-FSs prevents comparison to the retention rates locally. In the broader literature, the excellent retention rates of RB-FSs may not be applicable to the patient base of HDFT-CDS and hence it is impossible to draw an explicit conclusion of the efficacy of GIC-FSs.

Whilst the majority of the research around the effectiveness of fissure sealants reports retention as an outcome, current evidence suggests that full retention is not an accurate prediction of the effectiveness of fissures sealants as caries preventive measures, especially for GIC-based sealants [10, 17]. Therefore, although the findings from this case series provide interesting information regarding retention, further information would be required to see if the sealants carry out their primary purpose of prevention. As a secondary care unit, CDS-NY tends to discharge the majority of our patients that require general anaesthetic back to the care of their general dental practitioners following a single course of treatment, and therefore are unable to conduct long-term follow-up to identify patterns in caries prevention and fissure sealants.

As part of this case series, retention was only recorded as a binary measure, partial loss was not determined or assessed. Although some evidence suggests that partial loss of GIC can still be effective as the opening of the fissures is still sealed and fluoride is still being released [22]. To ascertain this would require a longer follow-up. We are also limited by the time for reviews: all FSs included in this case series were assessed and followed up for less than a year. As mentioned previously, within HDFT-CDS, it is difficult to follow up most patients long term.

### Generalisability

The results were derived from a local Service Evaluation and as such are utilised to inform local decision-making rather than make general recommendations. Furthermore, the HDFT-CDS sees patients with complex medical and social needs that are unable to accept treatment at general dental practices. As such, although the cases reviewed were in line with the general literature, the results are unlikely to be applicable to other clinical environments. The patients included in this case series were paediatric patients who assessed as high caries risk and were going for extractions under general anaesthetic. It is arguable that, where longer-term review is possible, clinical monitoring may present a valid alternative to RB-FS and GIC-FS and hence generalisation to other clinical processes is not recommended.

## Conclusion

Although the restrictions due to the pandemic have eased, this paper supports and adds to the existing evidence base on the efficacy of GIC-FS applications if RB-FSs are not achievable within the local setting and context. It further suggests that the appropriateness of GIC-FS should be considered in cases where broader limitations, such as clinical demands, time constraints, or national restrictions to practice are present.
